# Multiple Skeletal Muscle Metastases from Colon Carcinoma Preceded by Paraneoplastic Dermatomyositis

**DOI:** 10.1155/2013/392609

**Published:** 2013-07-24

**Authors:** Matteo Landriscina, Assunta Maria Teresa Gerardi, Alberto Fersini, Sergio Modoni, Luca Pio Stoppino, Luca Macarini, Francesca Sanguedolce, Pantaleo Bufo, Vincenzo Neri

**Affiliations:** ^1^Medical Oncology Unit, Department of Medical and Surgical Sciences, University of Foggia, Viale Pinto, 1-71100 Foggia, Italy; ^2^General Surgery Unit, Department of Medical and Surgical Sciences, University of Foggia, Viale Pinto, 1-71100 Foggia, Italy; ^3^Nuclear Medicine Unit, Riuniti Hospital, Viale Pinto, 1-71100 Foggia, Italy; ^4^Radiology Unit, Department of Clinical and Experimental Medicine, University of Foggia, Viale Pinto, 1-71100 Foggia, Italy; ^5^Pathology Unit, Department of Clinical and Experimental Medicine, University of Foggia, Viale Pinto, 1-71100 Foggia, Italy

## Abstract

Skeletal muscle metastases are very rare events in colorectal carcinoma. By contrast, dermatomyositis is an idiopathic inflammatory myopathy with characteristic cutaneous manifestations and a well-recognized association with several human malignancies and, among others, colorectal cancer. Here, we report the case of a 71-year-old woman with paraneoplastic dermatomyositis followed by the development of a metastatic colon cancer. Interestingly, this patient developed multiple skeletal metastases which were preceded by the worsening of systemic symptoms of dermatomyositis. This observation suggests that, while muscle tissue is usually resistant to the development of tumor metastases, the inflammatory and immune response which characterizes and boosts paraneoplastic myopathy may represent a favorable soil for tumor cell invasion and metastasization to skeletal muscles.

## 1. Introduction

Colorectal carcinoma is a very common malignancy in humans, and the liver is the most frequent site where colorectal cancer metastases occur, followed by lung, peritoneum, and bone. Skeletal muscle metastases are very rare [1], even though previous studies suggest that the muscle tissue may be occasionally involved at the late stage of colorectal carcinoma and usually as a part of disseminated disease [2]. Dermatomyositis (DM) is an uncommon inflammatory myopathy with characteristic rash accompanying, or more often preceding, muscle weakness [3, 4]. It is strongly associated with malignancy, which is diagnosed in about 24% of DM patients above the age of 50. Malignant disease may occur before the onset of myositis, concurrently, or afterward [4, 5]. The most commonly observed cancers in patients with DM are breast and gynecological cancers among women, lung cancer among men, and gastrointestinal malignancies in both sexes [3, 6].

## 2. Case Report

In May 2007, a 71-year-old woman was diagnosed with DM because of heliotropic rash over the upper eyelids, edema and erythema at the face and the upper extremities, skeletal muscle pain, and progressive weakness of the proximal muscles of her upper and lower limbs. Laboratory tests showed significant elevation of creatine kinase (3.7 times the normal levels) and anti-Jo1 antibodies positivity. Electromyography findings indicated myositis of the proximal muscles, and the muscle biopsy showed perimysial inflammation. She had been a heavy smoker until the age of fifty (more than 20 cigarettes per day), not addicted to alcohol, and she had a history of hypertensive cardiomyopathy. The patient was treated with systemic steroid therapy with good control of muscle pain and weakness and partial remission of skin rash. In July 2008, she was diagnosed with a carcinoma of the right colon with locoregional lymph node involvement and three liver metastases, all localized in the left lobe. Tumor genetic evaluation showed the presence of the BRAF V600E mutation. She underwent right hemicolectomy, subsequent systemic neoadjuvant chemotherapy with oxaliplatin and capecitabine (XELOX regimen) for 4 cycles with significant shrinkage of liver nodules and, in February 2009, resection of hepatic metastases. Systemic chemotherapy with the same agents was administered for additional 4 cycles after radical hepatectomy. A complete remission of clinical and laboratory signs of DM was also observed upon liver surgery.

**Figure 1:**
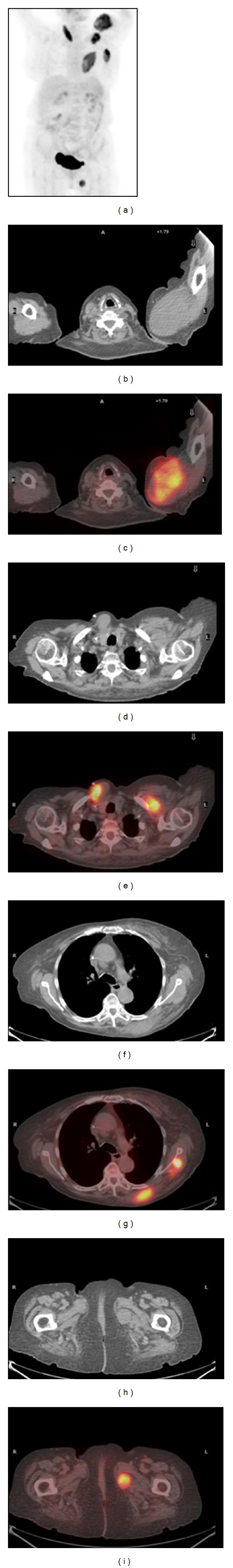
(a) Whole body PET scan showing multiple muscle metastases. ((b)–(i)) CT ((b), (d), (f), and (h)) and combined CT/PET ((c), (e), (g), and (i)) scan images showing multiple metastases in deltoid ((b) and (c)), sternocleidomastoid ((d) and (e)), trapezius and infraspinatus ((f) and (g)), and adductor magnus ((h) and (i)) muscles.

**Figure 2:**
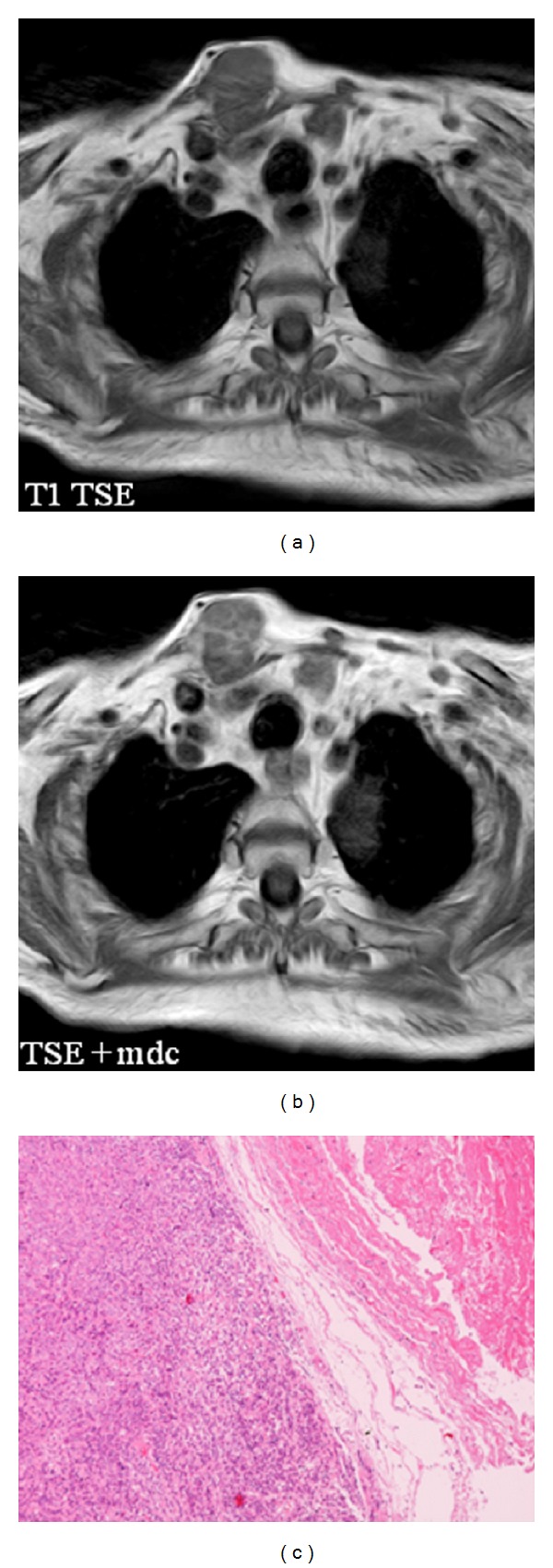
(a) and (b) MRI pre- (a) and postcontrast (b) scans showing a metastatic lesion in the right sternocleidomastoid muscle. (c) Hematoxylin and eosin staining of the sternocleidomastoid muscle lesion biopsy showing a metastasis from a poorly differentiated carcinoma (magnification: 100-fold).

The patient had a relapse-free survival of 7 months, until September 2009, when a computed tomography (CT) scan revealed multiple lung metastases. A parallel recurrence of facial erythema, skeletal muscle pain, and weakness of the proximal muscles was observed. Laboratory tests exhibited also elevation of creatine kinase levels and anti-Jo1 antibodies. A 2nd line chemotherapy with 5-fluorouracil, folinic acid, and irinotecan (FOLFIRI regimen) was administered for 5 months with complete remission of lung metastases and DM clinical and laboratory signs. In June 2010, the PET/CT scan exhibited the appearance of multiple skeletal metastases (Figure 1), with the major lesion being localized in the right deltoid muscle (Figures 1(b) and 1(c)). This finding was confirmed by the magnetic resonance imaging (MRI) of the neck showing a metastasis in the right sternocleidomastoid muscle (Figures 2(a) and 2(b)) and the fine needle biopsy of this metastatic lesion (Figure 2(c)). The progression of disease observed in June 2010 was preceded by the worsening of DM symptoms and signs; besides the patient was still under steroid therapy. Based on these lines of evidence, a 3rd line chemotherapy with 5-fluorouracile, folinic acid, and oxaliplatin (FOLFOX6 regimen) was administered for 3 cycles. The treatment was discontinued due to traumatic femur fracture, with subsequent rapid progression of disease and patient's death.

## 3. Discussion

The resistance of muscle tissues to the development of tumor metastases is a well-known clinical phenomenon [7]. Indeed, several mediators released by muscle cells may account for the rarity of metastatic nodules in this tissue, including cytokines with antitumor activity, such as TNF*α*, TGF*β*, lymphocyte infiltrating factor, interferon *γ*, lactic acid, and proteolytic enzymes like plasminogen activator inhibitor [7]. In this context, it has been suggested that small molecules present in conditioned medium of muscle cells and, among others, natural agonists of A3 adenosine receptor exert an inhibitory effect on the growth of several human tumor cell lines [7, 8]. Furthermore, muscle sarcolemma exerts a role of physical barrier against tumor cells [9]. In this case report, it is intriguing that skeletal muscle metastases, which represent a very rare event in human colorectal cancer [1], were preceded by prominent clinical signs of DM. Indeed, while DM is a paraneoplastic syndrome occasionally associated with colon carcinoma [3, 6], the molecular mechanism responsible for its development depends on the cross-reactivity between antigens expressed by cancer cells and regenerating fibers [3]. Furthermore, several lines of evidence suggest that inflammation and activation of the immune response represent favorable microenvironments for tumor cell invasion and dissemination [10, 11]. Thus, it is intriguing to speculate that the inflammatory and the immune responses that boost paraneoplastic DM may provide a favorable milieu for tumor cell invasion and metastasization to skeletal muscles, likely reverting the hostile environment that in nature characterizes muscle tissues. However, this hypothesis needs to be further explored in experimental tumor cell models.

## References

[1] Attili VSS, Rama Chandra C, Dadhich HK, Sahoo TP, Anupama G, Bapsy PP (2006). Unusual metastasis in colorectal cancer. *Indian Journal of Cancer*.

[2] Brennan JL (1971). Metastatic tumours of the diaphragm. *British Journal of Surgery*.

[3] Zahr ZA, Baer AN (2011). Malignancy in myositis. *Current Rheumatology Reports*.

[4] Levine SM (2006). Cancer and myositis: new insights into an old association. *Current Opinion in Rheumatology*.

[5] Hill CL, Zhang Y, Sigurgeirsson B (2001). Frequency of specific cancer types in dermatomyositis and polymyositis: a population-based study. *The Lancet*.

[6] Shah KR, Boland CR, Patel M, Thrash B, Menter A (2013). Cutaneous manifestations of gastrointestinal disease: part I. *Journal of American Academy Dermatology*.

[7] Bar-Yehuda S, Barer F, Volfsson L, Fishman P (2001). Resistance of muscle to tumor metastases: a role for A3 adenosine receptor agonists. *Neoplasia*.

[8] Djaldetti M, Sredni B, Zigelman R, Verber M, Fishman P (1996). Muscle cells produce a low molecular weight factor with anti-cancer activity. *Clinical and Experimental Metastasis*.

[9] Zacks SI, Vandenburgh H, Sheff MF (1973). Cytochemical and physical properties of myofiber external lamina. *Journal of Histochemistry and Cytochemistry*.

[10] Zampieri S, Valente M, Adami N (2010). Polymyositis, dermatomyositis and malignancy: a further intriguing link. *Autoimmunity Reviews*.

[11] Mendoza M, Khanna C (2009). Revisiting the seed and soil in cancer metastasis. *International Journal of Biochemistry and Cell Biology*.

